# Prostate cancer screening: Knowledge, attitudes and practices in a sample of men in Italy. A survey

**DOI:** 10.1371/journal.pone.0186332

**Published:** 2017-10-12

**Authors:** Marianna Morlando, Concetta Paola Pelullo, Gabriella Di Giuseppe

**Affiliations:** Department of Experimental Medicine, University of Campania “Luigi Vanvitelli”, Naples, Italy; Medizinische Universitat Innsbruck, AUSTRIA

## Abstract

**Background:**

The aim of this study was to evaluate the knowledge, attitudes and behaviors towards prostate cancer and its prevention.

**Methods:**

A cross-sectional survey was conducted on a random sample of 625 fathers of students attending eight public schools. The self-administered questionnaire included questions on: socio-demographic characteristics, personal and familiar medical history of prostate cancer, knowledge about prostate cancer and the prostate-specific antigen (PSA) test, perception of risk towards prostate cancer, perception of the benefits of having a PSA-test, willingness to receive the PSA-test and sources of and needs of information regarding prostate cancer and the PSA-test.

**Results:**

72.7% of respondents had heard about the PSA-test and 51.1% of those had heard about it through their physicians. This knowledge was higher: in men with older age, in those that had a higher level of education, in those who had a relative with prostrate problems or prostate cancer and in those with prostate problems. Perceived personal risk of contracting prostate cancer was associated with a higher level of education, in those who had received information about prostate cancer from a physician and in those with prostate problems. Only 29.6% of men had undergone a PSA-test and 59.4% were willing to do so in the future. The significant predictors of the willingness to receive a PSA-test were the belief that the PSA-test was useful, the perception of not very good personal health status, and need of additional information about the PSA-test.

**Conclusion:**

Respondents have a moderate knowledge about prostate cancer and a good propensity to undergo the PSA-test. Therefore, it would be necessary to increase information on the risks of prostate cancer and the benefits of prostate cancer prevention.

## Introduction

Prostate cancer is the most common malignancy occurring in men, particularly, it is the second most common of all diagnosed cancers and represents the sixth leading cause of cancer death worldwide with 1,111,700 new cases of prostate cancer diagnosed and 307,500 deaths in 2012 [1]. In Europe, there were 400,364 new cases and regarding mortality there were 92,328 deaths in 2012 [2]. In 2015, it was estimated that 3,037,127 Italians had been previously diagnosed with cancer and 398,708 were previously diagnosed with prostate cancer [3].

Due to the aging population and population growth, the expected numbers will increase in forthcoming years. Thus, prevention and early detection has immense public health importance. Currently, there is no scientific consensus on effective strategies to reduce the risk of prostate cancer. The prostate-specific antigen (PSA) test is widely used to screen for prostate cancer but its use is controversial for several reasons [4–7].

In 2012 the United States Preventive Service Task Force [8] suggested discontinuing PSA-based screening for prostate cancer (PCa) screening in all men. In contrast, the American Urological Association [9] recommends that PSA screening, in conjunction with a digital rectal examination, be offered to asymptomatic men aged 40 years or older who wish to be screened and the American Cancer Society [10] emphasizes informed decision making for prostate cancer screening.

All protagonists in the public controversy agree that men should make an informed decision about whether or not to undergo PSA screening and, hence, need to be fully appraised of the arguments for and against it [11–12].

Given the complexity of the issues regarding prostate cancer screening, experts recommend that men receive information on the benefits and risks of screening before making decisions. In literature, there is limited experience shared regarding knowledge,[13–16] attitudes [13–18] and behaviors [18–19] towards prevention of prostate cancer.

Therefore, the aim of this study is to assess knowledge, attitudes and behaviors regarding prevention of prostate cancer and determinants associated with these outcomes, in a sample of adult men in southern Italy.

## Materials and methods

A cross-sectional survey design was used in this study. The eligible study population included a random sample of 750 fathers of students between the ages of 14–18 attending eight public schools in the area of Naples, in the south of Italy, during the period from January to April 2011.

The number of fathers sampled in this study was calculated before study initiation, considering a prevalence of 42.8% of adults that undergo the PSA test, using a confidence interval of 95%, a margin of error of 5%, and an expected response rate of 50%. So, it was estimated a total number of 661 fathers were to be sampled.

Before giving their approval, the directors of each school selected received a letter inviting the school to help cooperate by contacting the fathers of their student body to participate. It explained the objectives and methodology of study.

Students were asked to take home a sealed envelope which enclosed: an introductory letter, an informed consent form, a two-page questionnaire, and a self-addressed envelope to return the survey. The returned envelopes were collected by a contact person at the school and delivered to the organizers of the survey.

The letter included: 1) information such as the organization behind the study; 2) the contact name and address of the researcher; 3) details of how and why the responders was selected; 4) the aims of the study.

Participants were assured that participation in the survey was voluntary and anonymous. They were assured of the confidentiality of the information provided and that all data was to be processed anonymously. Moreover, an envelope to facilitate the return of the completed questionnaire was made available. All participants provided written informed consent at the beginning of the survey prior to answering any question by reading the consensus form.

Data was collected using a self-administered anonymous structured questionnaire, divided into seven major parts.(S1 File) The survey questionnaire sought information about socio-demographic characteristics, personal and familiar medical history of prostate cancer, knowledge about prostate cancer and the PSA-test, perception of risk towards prostate cancer, perception of the benefits of having a PSA-test, willingness to receive the PSA-test, and sources of and needs of information regarding prostate cancer and the PSA-test.

The socio-demographic section focused on the personal characteristics of the sampled men, such as age, marital status, educational level and occupation. Then their self-reported health status and their personal or familiar history of prostate cancer were assessed. Knowledge was tested by asking respondents to answer questions regarding risk factors and screening tests of prostate cancer. Their beliefs about prostate cancer and the PSA-test were measured on a 3-point Likert-type scale with options agree, uncertain, and disagree. Attitudes about risk of developing prostate cancer were measured with a 10-point Likert scale ranging from 1 (not worried) to 10 (extremely worried); moreover, their opinion about the perceived benefits of having the PSA-test was evaluated with a 10-point Likert scale. The respondents' behaviors were investigated by asking them to indicate whether or not: 1) they had received a health checkup for prostate problems; 2) the physician discussed the PSA-test with them and its utility; 3) they had undergone a PSA-test; 4) their willingness to undergo a PSA-test.

Finally, sources of information on prostate cancer and the PSA-test were evaluated by including a list of options with the possibility to indicate more than one source; the question about educational needs included a response in the ‘yes' and ‘no' format.

Before the initiation of the study, the questionnaire was reviewed for content and comprehensibility by a sample of 50 men to identify whether respondents would understand the questions and instructions, and whether the meaning of the questions was the same for all respondents. A group of experts reviewed the format and content of the items, as well as the content validity of the instrument as a whole. The internal reliability was assessed using Cronbach’s α.

The Ethics Committee of the Second University of Naples (renamed, in 2016, University of Campania “Luigi Vanvitelli”) approved this study.

### Statistical analysis

There were four outcomes of interest: a) had heard about the PSA-test (no = 0, yes = 1), (Model 1) b) perception of risk of developing prostate cancer (no = 0, yes = 1), (Model 2) c) had had a PSA-test (no = 0, yes = 1), (Model 3) d) willingness to receive a PSA-test (no = 0, yes = 1) (Model 4). In all models, the independent variables included were the following: age (continuous, in years), marital status (other = 0, married = 1), occupation (unemployed = 0, employed = 1), education level (three categories: middle school or lower = 1, high school = 2, college degree or higher = 3), perception of personal health status (other = 0, good = 1), relatives with any prostrate problems/prostate cancer (no = 0, yes = 1), had had prostate problems (no = 0, yes = 1), felt the need of additional information about the PSA-test (no = 0, yes = 1).

The following variables were also included: physician as the source of information about the PSA-test (no = 0, yes = 1) in Models, 3 and 4; knowledge about prostate cancer (no = 0, yes = 1), knowledge about risk factors of prostate cancer (increased age>50 years and family history of prostate cancer) (no = 0, yes = 1), physician as source of information about prostate cancer (no = 0, yes = 1), need of additional information about prostate cancer (no = 0, yes = 1) in Models 2, 3 and 4; perception of risk of developing prostate cancer (no = 0, yes = 1), positive attitude towards the utility of the PSA-test for prostate cancer prevention (no = 0, yes = 1), had received a health checkup for prostate problems (no = 0, yes = 1), physician discussed the PSA-test (no = 0, yes = 1), in Models 3 and 4.

Bivariate appropriate analyses were tested to assess associations between potential explanatory variables and each outcome of interest. Afterwards, variables associated with each outcome of interest with a p-value ≤ 0.25 in bivariate analyses were introduced into multivariate regression model. Multivariate logistic regression analysis was conducted to determine significant independent characteristics associated the four outcome of interest. A stepwise procedure was used to obtain the final models. Variables were selected for the multivariate model using 0.2 for entry and 0.4 for exclusion. The results of multivariate regression analyses were reported as odds ratios (ORs) and 95% confidence intervals (CIs). All of the tests for significance were two-sided and p-values ≤ 0.05 were considered statistically significant. Analyses were carried out using Stata 10 [20].

## Results

The self-administered questionnaire was returned by a total of 625 subjects, with an overall response rate of 83.3%. Table 1 provides an overview of the respondents' socio-demographic characteristics. The average age of participants was 48.7 years (27–71 years), one-third had completed a high school education, the majority were married and employed, the mean value of perception of personal health status was 7.5, 11.6% reported a personal history of prostate problems, and 4.9% reported a familiar history of prostate cancer.

**Table 1:** Socio-demographic and selected information about the study population.

	N	%
*Age group (years)*	48.7±6.6(27–71)*	
<50	347	57.1
≥50	261	42.9
*Marital status*		
Married	508	83.7
Other	99	16.3
*Highest education level*		
No formal education	4	0.6
Elementary	70	11.4
Middle school	201	32.7
High school	213	34.6
College degree or higher	127	20.7
*Employment status*		
Employed	527	90.7
Unemployed	54	9.3
*Perception of personal health status*	7.5±1.6(1–10)*	
*Personal history of prostate cancer*		
Yes	72	11.6
No	553	88.4
*Familial history of prostate cancer*		
Yes	31	4.9
No	594	95.1

*Mean±Standard deviation (Range)

Numbers for each item may not add up to total number of study population due to missing values

Table 2 shows the answers to each question regarding knowledge about prostate cancer and the PSA-test. In particular, 82.1% reported having heard of prostate cancer before and 31.8% of those had heard about prostate cancer from a physician. Respondents answered correctly by indicating age>50 years (65.9%) and family history of prostate cancer (31.6%) as risk factors. 72.7% reported that they had heard about the PSA-test before and 51.1% of those had heard about it from a physician.

**Table 2:** Knowledge about prostate cancer and PSA test of the men who responded to the survey.

	N	%
*Had heard about prostate cancer as the most common cancer in men*		
Yes	513	82.1
No	112	17.9
Had heard of prostate cancer from a physician*		
Yes	163	31.8
No	350	68.2
Risk factors*		
Increased age (>50 years) (*True*)		
Yes	338	65.9
No	175	34.1
Family history of prostate cancer (*True*)		
Yes	162	31.6
No	351	68.4
*Had heard about the PSA test*^+^		
Yes	368	72.7
No	138	27.3
*Had heard about the PSA* test *by a physician*°		
Yes	188	51.1
No	180	48.9

* Only for those who reported that they had heard about prostate cancer

^+^ The number of participants responding to this question is 506

° Only for those who reported that they had heard about PSA test

The results of the logistic regression analysis revealed that the following factors were statistically significantly associated with knowledge about the PSA-test: older age (OR = 1.08; 95% CI 1.03-1-12), those who had a relative with problems/prostate cancer (OR = 2.77; 95% CI 1.4–5.5) and those who had a prostate problem (OR = 6.7; 95% CI 2.01–22.89). The education has also an impact on knowledge since respondents with a middle school or lower (OR = 0.2; 95% CI 0.1–0.43) and high school (OR = 0.35; 95% CI 0.16–0.76) were less knowledgeable compared to those with a college degree or higher. (Model 1 in Table 3).

**Table 3:** Results of univariate and multivariate analysis.

	Model 1		Model 2		Model 3		Model 4	
	Have heard of PSA-test		Perception of risk of developing prostate cancer		Have received PSA-test		Willingness to receive PSA-test	
	*Univariate*	*Multivariate*	*Univariate*	*Multivariate*	*Univariate*	*Multivariate*	*Univariate*	*Multivariate*
	*p*	OR (95% CI)	*p*	OR (95% CI)	*p*	OR (95% CI)	*p*	OR (95% CI)
Age	<0.0001	1.08 (1.03–1.12)	0.722	-	<0.0001	1.1 (1.05–1.15)	0.78	-
Marital status	0.763	-	0.439	-	0.971	-	0.276	-
Occupation	0.041	2.08 (0.92–4.69)	0.358	-	0.447	-	0.257	-
Education level								
Middle school or lower	<0.0001	0.2 (0.1–0.43)	0.073	1.56 (1.11–2.2)	<0.0001	0.58 (0.32–1.05)	0.711	-
High school		0.35 (0.16–0.76)		Backward elimination		Backward elimination		
College degree or higher		1*		1*		1*		1*
								-
Perception of personal health status	0.206	Backward elimination	0.039	0.65 (0.39–1.07)	0.002	0.55 (0.21–1.39)	0.201	0.53 (0.3–0.96)
Relatives with prostrate problems/prostate cancer	<0.0001	2.77 (1.4–5.5)	0.079	1.23 (0.8–1.89)	<0.0001	2.05 (1.05–3.99)	0.052	1.86 (1.01–3.42)
Had had prostate problem	<0.0001	6.7 (2.01–22.89)	<0.0001	2.3 (1.22–4.32)	<0.0001	Backward elimination	0.071	0.5 (0.19–1.33)
Need of additional information about PSA-test	0.583	-	0.48	-	0.259	-	<0.0001	2.6 (1.69–3.98)
Physician as source of information about PSA-test	-		-	-	<0.0001	2.74 (1.28–5.86)	0.298	-
Knowledge about prostate cancer	-	-	0.185	1.31 (0.84–2.04)	<0.0001	3.6 (1.2–10.83)	0.005	2.17 (1.34–3.53)
Knowledge about risk factors of prostate cancer	-	-	0.69	-	<0.0001	2.24 (1.18–4.24)	0.044	Backward elimination
Physician as source of information about prostate cancer	-	-	0.003	1.74 (1.15–2.64)	<0.0001	0.53 (0.24–1.15)	0.337	-
Need of additional information about prostate cancer	-		0.498	-	0.08	1.72 (0.99–2.99)	<0.0001	Backward elimination
Perception of risk of developing prostate cancer	-	-	-	-	0.007	Backward elimination	0.036	1.45 (0.95–2.22)
Positive attitude towards the utility of PSA-test for prostate cancer prevention	-	-	-	-	0.304	-	0.003	1.78 (1.12–2.84)
Had received a health checkup for prostate problems	-	-	-	-	<0.0001	3.1 (1.65–5.83)	0.754	-
Physician discussed the PSA-test	-	-	-	-	<0.0001	11.3 (6.01–21.35)	0.378	-

* Reference category in multivariate analysis

The mean value of the perceived personal risk of contracting prostate cancer, on a scale from 1 to 10, was 5.5. More than half (51.2%) reported that they were at risk of prostate cancer by responding from 6 to 10 in the question. To determine which factors were related to a participant's perceived risk of developing prostate cancer a multivariate logistic regression was performed, and the following factors were statistically significantly associated: middle school or lower educational level compared to those with a college degree or higher (OR = 1.56; 95% CI 1.11–2.2), those who had received information about prostate cancer by a physician (OR = 1.74; 95% CI 1.15–2.64), and those who had a prostate problem (OR = 2.3; 95% CI 1.22–4.32) (Model 2 in Table 3).

With regard to the behaviors, only 29.6% had received a PSA-test. Respondents with: older age (OR = 1.1; 95% CI 1.05–1.15), those who had discussed the PSA-test with a physician (OR = 11.3; 95% CI 6.01–21.35), those who had received a health checkup for prostate problems (OR = 3.1; 95% CI 1.65–5.83), those who had received information about the PSA-test by a physician (OR = 2.74; 95% CI 1.28–5.86), those who had heard about risk factors of prostate cancer (OR = 2.24; 95% CI 1.18–4.24), those who had heard about prostate cancer (OR = 3.6; 95% CI 1.2–10.83), and those who had a relative with prostate problems/prostate cancer (OR = 2.05; 95% CI 1.05–3.99), or were more likely to undergo a PSA-test (Model 3 in Table 3).

Among those that had undergone the PSA-test, 71.7% had had the test at least within the last year and 52.9% were recommended by a physician. The other reason for having the PSA-test were feeling at risk (12.4%) and participation in prevention programs. The most common reasons for not having a PSA-test were not feeling at risk (41.1%) and not having time (27.3%). (Table 4)

**Table 4:** PSA test history and the willingness to receive it.

	N	%
*PSA test history*		
Yes	185	29.6
No	440	70.4
PSA-test in the past 1 year*		
Yes	109	71.7
No	43	28.3
*Reasons for not doing the PSA test* *^§^		
I do not feel at risk	181	41.1
I have not time	120	27.3
I'm afraid of discovering prostate cancer	52	11.8
It has not been recommended	43	9.8
The test is not useful	23	5.2
Other reasons	66	15
*Reasons for doing the PSA test* *^§^		
I was recommended by	104	56.2
*Physician*	55	52.9
*Other*	49	47.1
I want to check for prostate cancer	54	29.2
I feel at risk	23	12.4
I have participated in prevention programs	19	10.3
*Willingness to receive a PSA test*°^+^		
Yes	257	59.4
No	176	40.6
*Reasons for unwillingness to receive PSA test*^§^°		
I do not feel at risk	112	63.6
I'm afraid of discovering prostate cancer	29	16.5
It has not been recommended	18	10.2
The test is not useful	16	9.1
*Reasons for willingness to receive PSA test*^§^°		
I want to discover prostate cancer	131	51
If recommended by physician	113	44
I feel at risk	18	7

*Only for those who received PSA test

°Only for those who did not receive PSA test

^§^Respondents could have selected more than one response

^+^The number of participants responding to this question is 433

Furthermore, as regard the willingness to receive the PSA-test as a main screening test for prostate cancer prevention, approximately little more than half (59.4%) responded ‘‘yes” on being asked about willingness to receive the PSA-test. The results showed that those who were more willing to undergo a future PSA-test were those who needed additional information about the PSA-test (OR = 2.6; 95% CI 1.69–3.98), those who had heard about prostate cancer (OR = 2.17; 95% CI 1.34–3.53), those who had a positive attitude towards the utility of the PSA-test for prostate cancer prevention (OR = 1.78; 95% CI 1.12–2.84), those who had a relative with prostate problems/prostate cancer (OR = 1.86; 95% CI 1.01–3.42) and those who considered themselves as not having an excellent personal health status (OR = 0.53; 95% CI 0.3–0.96). (Model 4 in Table 3).

When the willingness to receive the PSA-test was investigated, the main reasons were prevention (51%), if recommended by physician (44%), and feeling at risk (7%).

In terms of sources of information about prostate cancer, the most frequent were television/newspapers (49.6%), physicians (36.2%), family (31.4%) and the internet (11.4%). With regards to the PSA test, the most frequent source of information was physicians (54.4%) followed by television/newspapers(35.8%) and family (23.4%). 57.9% and 36.3% indicated, respectively, that they would like more information about prostate cancer and the PSA-test.

## Discussion

Our study provides an overview about contemporary opinions on prostate cancer screening. The PSA-test has received negative press in recent years, for this reason the controversy surrounding screening continues. Since a reduction of incidence of disease through effective primary prevention or from the use of pharmacological treatments is not expected, at least in the short term, secondary prevention with PSA testing appears to remain the most appropriate instrument. Moreover, decisions about prostate cancer screening should be based on the preferences of an informed patient.

In this study, the majority of men had an adequate knowledge about prostate cancer (82.1%). A similar value has also been observed in another study conducted in Jamaica, with 96% of men responding correctly to questions about prostate cancer [16]. By contrast, in comparison with other studies from different countries, this rate was better than that which has been reported in South Africa (45.7%) among men attending an urologic outpatient clinic,[21] and in Uganda (54.1%) [22]. Regarding knowledge about the PSA-test, we found that 72.7% had heard of it before. This value is higher than the 52.1% reported in a survey conducted in Uganda [22].

Amongst the risk factors for developing prostate cancer there are being over the age of 50 and having a family history of the disease. 65.9% of the respondents correctly identified age>50 years and only 31.6% family history of prostate cancer as risk factors for this type of cancer. Regarding family history of prostate cancer, a similar finding has been observed in a South African study where 32.3% of respondents knew, as a possible cause of prostate cancer, family inheritance [21]. This rate was the lowest compared with previously mentioned studies, where this knowledge was, respectively, 84% and 61.4%[16,22]. By contrast, a considerably lower value has been observed in a study conducted in Burkina Faso in a sample of black African men in which only 4.2% knew heredity as a risk factor of prostate cancer[23].

In our sample, only 29.6% had received a PSA-test. This finding is similar to another study conducted in South Africa where men who had received a PSA-test were only 28.3%[21]. In our study, amongst the men that had received a PSA-test, about half were recommended to do so by a physician (52.9%). A similar finding has been observed in a study conducted in the United States, which found that men that had spoken with a physician about the PSA-test were more likely to be screened[7].

Another important finding is attitudes towards the PSA-test. About 60% of respondents expressed their willingness to receive a PSA-test. This intention was similar when compared with another study conducted in Spain (57.9%)[24].

The major sources of information about the PSA-test were physicians, television/newspapers and family. Men who had received information by a physician were more likely to know about and to receive a PSA-test. This finding can be compared with other studies. For example, Carrasco-Garrido *et al* [24] found that men who had received information from healthcare workers had a higher probability to know about and to receive a PSA-test. Also in another study conducted in the United States of America, the most frequent source of information were physicians (86%) and mass media (62%). By contrast, in a previously cited study,[22] only 12.3% of respondents reported that physicians informed them about screening for prostate cancer.

The benefits arising from the activation of a screening program are still uncertain and not supported by sufficient scientific evidence. Indeed, there may be psychological repercussions and on the quality of life after treatment. For these reasons patients (by age, risk factors and life expectancy) should be properly informed by physicians about the advantages and disadvantages of the test. Therefore, the role of physicians remains essential in involving patient choice. This is already evidenced by our study, in which the physician is an important source of information (54.4%) for the PSA-test and those who have received a PSA-test, in 52.9% of men they underwent the PSA-test because it was recommended by a physician.

This study has some potential limitations. First, this study was based on a cross-sectional design and so there is not a clear association between the dependent and independent variables. Second, this survey was based on a self-administrated questionnaire and the participants could describe their perceived idea of correct behavior and not their real behavior. Thirdly, men responded to the questionnaire at home and it is possible that some respondents before answering might have sought other information on the issue.

## Conclusion

The present study provided information about knowledge, attitudes and behaviors about prostate cancer prevention in the general population in Italy and supports the need of adequately informing men about the harms and benefits of the PSA-test, so that they make an informed decision on the test. Therefore, it would be interesting to conduct another study to investigate physicians' behaviors and attitudes towards the PSA-test. The results of this study could be used to improve and promote the use of preventive services.

## Supporting information

S1 File English questionnaire. (DOCX)

S2 File Italian questionnaire. (DOC)
