# Supplementary material for: Prostate cancer screening: Knowledge, attitudes and practices in a sample of men in Italy. A survey
Source: PLoS One. 2017 Oct 12;12(10):e0186332. doi: 10.1371/journal.pone.0186332 (PMC5638517; doi:10.1371/journal.pone.0186332)
Supplement: S1 File — (DOCX) [file pone.0186332.s001.docx]

**A.** **SOCIO-DEMOGRAPHIC INFORMATION**

**A1.** How old were you on your last birthday? _______

**A2.** What is your marital status? married single (never married) other ___________

**A3.** What is your highest level of education?__________________________________________________

**A4.** What is your occupation?_____________________________________________________________

**A5.** On a scale from 1 to 10 how would you classify your current state of health?

(1 bad; 10 very good)________

**A6.** Have you or any of your relatives (father, brothers, etc.) ever had prostate problems?

no yes, I've had (specify the type of problem) ______________________________

yes, indicate who _______________ at what age _____ and what he had: ________

**B. KNOWLEDGE**

**B1.** Prostate cancer is the most common malignancy occurring in men. Have you ever heard of it? no (go to question **C**1.) yes, from whom? mass media physician internet friends/family other____

**B2.** What may favor the onset of prostate cancer? **(more than one answer is possible)**

high-fat diet smoking older than 50 years

alcohol obesity family history

number of sexual partners other_______________________________________________

**B3.** At what age are men more at risk of developing cancer of the prostate?______

**B4.** What can prevent the onset of prostate cancer? **(more than one answer is possible)**

meat physical activity low fat diet, vitamin D/E

butter fruit and vegetables (at least 5 servings per day) other_________

**B5.** The PSA (Prostate-Specific Antigen) test is a blood test that allows you to locate cancer before the symptoms occur. Have you ever heard of it?

no yes, from whom? mass media physician internet friends/family other____

**C. ATTITUDES**

**For each of the following statements indicate whether you are in agreement, uncertain or disagree agreement uncertain disagree**

**C1.** The possibility of developing a prostate cancer increases with age

The PSA test is an invasive laboratory test

Men without symptoms, with more than 50 years of age, must undergo the PSA test

**C2.** On a scale from 1 to 10 how much are you worried about developing prostate cancer? (1 not worried; 10 very worried)________

**C3.** On a scale from 1 to 10 how useful is the PSA test to detect cancer before the symptoms occur? (1 not useful; 10 very useful)_______

**D. PRACTICES**

**D1.** Have you ever gone to the physician and/or an urologist for prostate problems?

no

yes, physician (how many times?________) yes, urologist (how many times?________)

**D2.** Did the physician and/or urologist tell you about the PSA test? no (**go to the question D4**.)

yes, physician, when I was ___ years old yes, urologist, when I was ___ years old

**D3.** Has a physician and / or urologist informed you about the advantages and disadvantages of having the PSA test?

no yes

**D4.** Have you ever had the PSA test?

no yes, when the last time?_________

| Why? **(more than one answer is possible)** | Why? **(more than one answer is possible)** |
| --- | --- |
| I was advised against it  I do not feel at risk  Lack of time  Afraid of discovering prostate cancer  It's not useful  Other_____________________________________ | I was advised **(who?_________)**  I feel at risk  I have participated in prevention programs  To detect prostate cancer before symptoms occur  Other _______________________________________  **(go to the question E1.)** |

**D5.** Would you receive the PSA test? **(1 absolutely no; 10 absolutely yes)**

1 2 3 4 5 6 7 8 9 10

| Why? **(more than one answer is possible)** | Why? **(more than one answer is possible)** |
| --- | --- |
| The physician has dissuaded me  I do not feel at risk  Afraid of discovering prostate cancer  It's not useful  Other___________________________________ | After talking to my doctor  I feel at risk  To detect prostate cancer before symptoms occur  Other______________________________________ |

**INFORMATION**

**E1.** Have you ever received information on prostate cancer? **(more than one answer is possible)**

no yes, from who? mass media physician internet friends/family other___________

**E2.** How useful do you think, on a scale of 1 to 10, the information was that you received on prostate cancer? (1 not useful; 10 very useful)_______

**E3.** Do you need more information about prostate cancer? no yes

**E4.** Have you ever received information about the PSA test? **(more than one answer is possible)**

no yes, from whom? mass media physician internet friends/family other_____________

**E5.** How useful do you think, on a scale of 1 to 10, the information you received on the PSA test was? (1 not useful; 10 very useful)_______

**E6.** Do you feel you need more information about the PSA test? no yes
