# Supplementary material for: Prostate cancer screening: Knowledge, attitudes and practices in a sample of men in Italy. A survey
Source: PLoS One. 2017 Oct 12;12(10):e0186332. doi: 10.1371/journal.pone.0186332 (PMC5638517; doi:10.1371/journal.pone.0186332)
Supplement: S2 File — (DOC) [file pone.0186332.s002.doc]

**A.** **INFORMAZIONI ANAGRAFICHE ED ANAMNESTICHE**

**A1.** In che anno è nato? ____ **A2.** Qual è il Suo stato civile?  sposato  celibe  altro___

**A3.** Qual è il più elevato titolo di studio che ha conseguito?____________________________

**A4.** Qual è la Sua attuale attività lavorativa?______________________________________________________________

**A5.** In una scala da 1 a 10 come classificherebbe il Suo stato di salute attuale? **(indicare con 1 cattivo e con 10 ottimo) ____**

**A6.** Lei o qualcuno dei Suoi parenti (padre, fratelli, etc.) ha mai avuto problemi alla prostata?

 no  sì, io ed ho avuto **(specificare il tipo di problema)** ____________________________________

 sì, indichi chi_______________a che età_____e cosa ha avuto_________________________

**B. CONOSCENZE**

**B1.** La neoplasia della prostata è uno dei più comuni tumori tra gli uomini. Ne ha mai sentito parlare?

 no (vada alla domanda **C**1.)  sì, da chi?  TV/giornali  medici  internet  amici/familiari  altro_____

**B2.** Cosa può favorire l’insorgenza della neoplasia della prostata? **(indicare anche più di una risposta)**

 dieta ricca di grassi  fumo  età superiore ai 50 anni

 alcool  obesità  familiarità

 numero di partner sessuali  altro__________________________________________________________

**B3.** A quale età gli uomini sono più a rischio di poter sviluppare la neoplasia della prostata? ________________________

**B4.** Cosa può prevenire l’insorgenza della neoplasia della prostata? **(indicare anche più di una risposta)**

 carne rossa  attività fisica  dieta povera di grassi  vitamina D/E

 burro  frutta e verdura (almeno 5 porzioni al giorno)  altro_____________

**B5.** Il PSA (Antigene Prostatico Specifico) test, è un esame del sangue che permette di individuare il tumore prima che i sintomi si manifestino. Ne ha mai sentito parlare?

 no  sì, da chi?  TV/giornali  medici  internet  amici/familiari altro_____

**C. ATTITUDINI**

**Per ciascuna delle seguenti affermazioni indicare se è in accordo, incerto o in disaccordo. D’accordo Incerto In disaccordo**

**C1.** La possibilità di sviluppare una neoplasia della prostata aumenta con l’età   

Il PSA test è un esame di laboratorio invasivo   

Gli uomini, senza sintomi, con più di 50 anni devono eseguire il PSA test   

**C2.** In una scala da 1 a 10 quanto è preoccupato di sviluppare una neoplasia della prostata? (1 indica non preoccupato e 10 molto preoccupato)_______

**C3.** Secondo Lei, in una scala da 1 a 10, quanto è utile il PSA test per individuare il tumore prima che i sintomi si manifestino? (indicare con 1 inutile e 10 molto utile)_______

**D. COMPORTAMENTI**

**D1.** E’ mai andato dal medico di base e/o da un urologo per problemi alla prostata?

 no

 sì, dal medico di base (quante volte?________)  sì, dall’urologo (quante volte?________)

**D2.** Il medico di base e/o l’urologo Le hanno parlato del PSA test?  no (**vada alla domanda D4**.)

 sì, il medico di base quando io avevo____anni  sì, l’urologo quando io avevo____anni

**D3.** E’ stato informato dal medico di base e/o dall’urologo sui vantaggi e gli svantaggi di effettuare il PSA test?  no  si

**D4.** Ha mai effettuato il PSA test?

 no  sì, quando l’ultima volta?_________

| Perché? **(indicare anche più di una risposta)** | Perché? **(indicare anche più di una risposta)** |
| --- | --- |
| mi è stato sconsigliato   non mi sento a rischio   mancanza di tempo   paura di scoprire la neoplasia della prostata   non è utile   altro_____________________________________ |  mi è stato consigliato **(indicare da chi_______________)**  ? mi sentivo a rischio   ho partecipato a programmi di prevenzione   per individuare la neoplasia della prostata prima che i sintomi si manifestino   altro________________________________________  **(vada alla domanda E1.)** |

**D5.** Farebbe il PSA test? **(1 indica assolutamente no e 10 assolutamente sì)**

1 2 3 4 5 6 7 8 9 10

| Perché? **(indicare anche più di una risposta)** | Perché? **(indicare anche più di una risposta)** |
| --- | --- |
| mi è stato sconsigliato dal mio medico   non mi sento a rischio   paura di scoprire la neoplasia della prostata   non è utile   altro ___________________________________ |  dopo aver parlato con il mio medico   mi sento a rischio   per individuare la neoplasia della prostata prima che i sintomi si manifestino   altro______________________________________ |

**INFORMAZIONI**

**E1.** Ha mai ricevuto informazioni in tema di neoplasia della prostata? **(indicare anche più di una risposta)**

 no  sì, da chi?  TV/giornali  medici  internet  amici/familiari  altro__________________

**E2.** Quanto ritiene utile, in una scala da 1 a 10, le informazioni che ha ricevuto in tema di neoplasia della prostata?

(1 indica non utile e 10 molto utile)_____

**E3.** Ha bisogno di altre informazioni sulla neoplasia della prostata?  no sì

**E4.** Ha mai ricevuto informazioni sul PSA test? **(indicare anche più di una risposta)**

 no  sì, da chi?  TV/giornali  medici  internet  amici/familiari  altro__________________

**E5.** Quanto ritiene utile, in una scala da 1 a 10, le informazioni che ha ricevuto sul PSA test?

**(1 indica non utile e 10 molto utile)_____**

**E6.** Ha bisogno di altre informazioni sul PSA test?  no  sì
